# Calcification and ecological depth preferences of the planktonic foraminifer *Trilobatus trilobus* in the central Atlantic

**DOI:** 10.1098/rsos.240179

**Published:** 2024-12-04

**Authors:** Stergios D. Zarkogiannis

**Affiliations:** ^1^ Department of Earth Sciences, University of Oxford, South Parks Road, Oxford OX1 3AN, UK

**Keywords:** planktonic foraminifera, ecology, calcification, shell architecture, X-ray micro-computed tomography

## Abstract

Understanding the controls behind the calcification and distribution of planktonic foraminifera in the modern ocean is important when these organisms are used for palaeoceanographic reconstructions. This study combines previously reported shell mass data with new shell geochemistry, light microscopy and X-ray micro-computed tomography analyses to dissect various parameters of *Trilobatus trilobus* shells from surface sediments, investigating the factors influencing their biometry. The goal is to understand which aspects of the marine environment are critical for the calcification and vertical distribution of this species. *Trilobatus trilobus* is found to produce larger, thinner and overall lighter shells in equatorial regions than in subtropical gyre regions, where the shells are up to 4% smaller, more than 60% thicker and approximately 45% heavier. The skeletal mass percentage together with other calcification metrics (shell weight and thickness) are found to depend primarily on ambient seawater salinity rather than carbonate chemistry. In line with their degree of calcification, on the basis of geochemically reconstructed apparent calcification depths, this group of organisms is found shallower in the water column at the Equator and the subtropical gyres, while its habitat deepens in between these regions at the extra-equatorial sites. Furthermore, it is demonstrated that in the (central) Atlantic, it occupies a density layer slightly below the salinity maximum isopycnal at various depths, presumably by adjusting its shell properties.

## 1. Introduction

Sedimentary planktonic foraminifera shells and their geochemical compositions are widely utilized as proxies of past oceanic conditions such as temperature, salinity, ocean stratification, atmospheric CO_2_ concentrations and biological productivity [1,2]. Considering that planktonic foraminifera constitute a major fraction (approx. 32–80%) of deep-sea carbonate sediments [3], understanding their life cycles and biomineralization strategies is critical for understanding the history of the marine carbon cycle and, hence, the mechanisms regulating atmospheric CO_2_ concentrations. However, the application of fossil shells of planktonic foraminifera to decipher past environmental changes requires individual species analyses [4]. Since the degree of planktonic foraminifera calcification is a function of calcification depth [5] and calcification depth varies spatially and among species, improved past carbonate budget estimates may require species-specific analyses.

*Trilobatus trilobus* is a shallow-dwelling (i.e. mixed-layer rather than (sub)thermocline) planktonic foraminifer morphospecies [6,7] that is common in (sub)tropical assemblages worldwide and has a long (from latest Oligocene/early Miocene to recent) stratigraphical range [8]. *Trilobatus trilobus* is part of the *Trilobatus sacculifer* plexus, which consists of four morphospecies (*T. trilobus*, *T. immaturus*, *T. quadrilobatus* and *T. sacculifer*) but has only one genetic type [9]. In the global ocean, pelagic calcium carbonate accumulation is confined between approximately 30° north and south of the Equator with higher rates in the Atlantic, where the accumulation zone extends between approximately 40° north and south [10]. In the Atlantic, the presence of *T. trilobus* is continuous from approximately 40° S to marginally north of 40° N [11]. By being the dominant species in the equatorial regions of all oceans [6] and having high abundance in all of the different (both oligotrophic and upwelling) low-latitude oceanographic regions [7], *T. trilobus* is thus not only useful for palaeoceanographic studies but also an important contributor to pelagic calcium carbonate production.

Planktonic foraminifera, such as *T. trilobus*, are relatively simple unicellular organisms that cannot actively swim (hence termed planktonic rather than nektonic); they can only control their buoyancy [2,12,13]. In recent years, the palaeoceanographic focus has been on foraminifera shell geochemistry; however, recent advances in X-ray micro-computed tomography (μCT) have allowed the detailed study of the physical and architectural characteristics of microfossils. In the present study, the geochemistry of core-top fossil shells is used to determine the apparent calcification depth (ACD) of *T. trilobus* in the central Atlantic along the Mid-Atlantic Ridge and is shown to be confined within the subtropical circulation cells. Furthermore, published shell weights were combined with novel μCT analysis data to determine various physical properties and traits of *T. trilobus* shells, including shell thickness, volume and bulk shell density (volume-normalized shell weights). These findings suggest that adult specimens, following the subsurface salinity maximum density horizon, adapt their shells to inhabit subsurface waters in the tropics and surface waters at the Equator and in the subtropical gyres.

## 2. Regional setting

**Figure 1.**
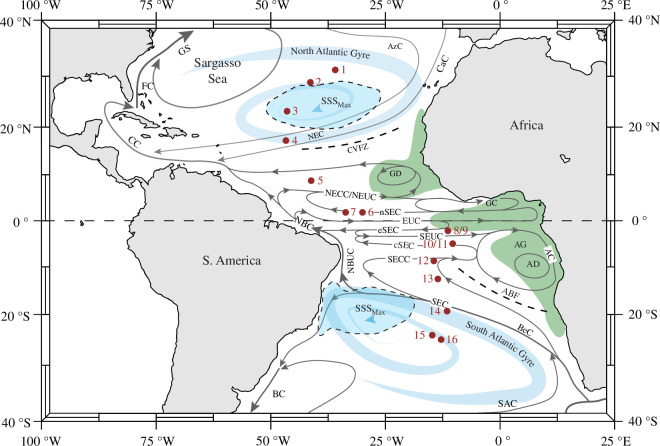
Locations of the core-top samples and schematic representation of the surface currents, subtropical gyres upwelling areas (in green) and major fronts of the Atlantic Ocean, many of which also operate at thermocline depths. ABF, Angola-Benguela front; AD/AG/AC, Angola Dome/Gyre/current; AzC, Azores current; BC, Brazil current; BG, Benguela current; CaC, Canary current, CC, Caribbean current; CVFZ, Cape Verde Frontal Zone; EUC, equatorial undercurrent; FC, Florida current; GD/GC, Guinea Dome/current; GS, Gulf stream, NBC, North Brazil current; NBUC, North Brazil undercurrent; NEC, North equatorial current; NEUC, North Equatorial Undercurrent; SAC, South Atlantic current; SEC, South equatorial current with its northern (nSEC), equatorial (eSEC) and central (cSEC) branches; SEUC, South equatorial undercurrent, SSS_max_, sea surface salinity maximum.

The current study is based on a core-top sample set from 16 locations along the Mid-Atlantic Ridge in the central Atlantic. More information about those samples can be found in table 1 and Zarkogiannis *et al*. [5]. At the near-surface, Atlantic circulation is forced by continental boundaries into closed cells and is primarily wind driven, with subtropical gyres at intermediate latitudes. The waters subducted in the subtropical gyres during the winter season are transported to the tropics, where they are upwelled to the surface in the eastern equatorial region forming the subtropical cells [17] (STCs) that are shallow meridional overturning cells (figure 1).

**Table 1.** Locations, water depths, sample levels and stratigraphic constraints of the studied core tops along the Mid-Atlantic Ridge. The ages of some samples were verified via radiocarbon dating, whereas the remaining samples were dated via oxygen isotope stratigraphy and carbonate content in various studies. strat. stands for stratigraphy and iso. strat. for isotope stratigraphy.

sample	latitude	longitude	water depth (m)	core depth (cm)	age	stratigraphy	reference
1. VM27-261	31.367	−35.983	3253	0	4.550 ± 0.035 ka	^14^C	Cléroux *et al*. [14]
2. VM19-308	29.017	−41.4	3197	4.5	4.520 ± 0.035 ka	^14^C	Cléroux *et al*. [14]
3. VM16-206	23.333	−46.483	3733	6.5	Holocene	iso. strat.	Cléroux *et al*. [14]
4. VM23-112	17.267	−46.75	2845	4.5	5.800 ± 0.086 ka	^14^C	This study
5. VM22-26	8.717	−41.25	3720	12–13	Holocene	% carbonate	Dai *et al*. [15]
6. RC13-189	1.863	−30	3233	4–5	0−2.5 kyr BP	^14^C strat.	Dai *et al*. [15]
7. RC13-188	1.817	−33.683	3451	2.5	4.478 ± 0.079 ka	^14^C	This study
8. RC24-10	−2.177	−11.252	3451	2.5	0−2.5 kyr BP	^14^C strat.	Broecker *et al*. [16]
9. RC24-11	−2.183	−11.25	3445	0	2.655 ± 0.035 ka	^14^C	Cléroux *et al*. [14]
10. RC24-16	−5.038	−10.192	3559	0	Holocene	iso. strat.	Cléroux *et al*. [14]
11. RC24-17	−5.05	−10.183	3559	0.5	2.635 ± 0.035 ka	^14^C	Cléroux *et al*. [14]
12. VM22-175	−8.767	−14.283	2950	0	Holocene	% carbonate	Cléroux *et al*. [14]
13. RC16-77	−12.652	−13.437	3404	6.5	Holocene	iso. strat.	Cléroux *et al*. [14]
14. VM16-36	−19.367	−11.433	3329	13–14	0−5 kyr BP	^14^C strat.	Dai *et al*. [15]
15. RC08-19	−24.3	−14.7	3636	10.5	Holocene	% carbonate	Cléroux *et al*. [14]
16. RC08-23	−25.15	−12.767	3338	9.5	Holocene	iso. strat.	Cléroux *et al*. [14]

Within the STCs, the upwelled water is modified by air–sea heat exchange and then advected back to the subtropics by poleward Ekman flows in the surface layer to complete the STC. The cells connect the subduction zones of the eastern subtropics of both hemispheres through equatorward boundary undercurrents with the equatorial undercurrent (EUC) [18]. The general east-to-west flow of surface currents in the tropical Atlantic allows cool nutrient-rich intermediate-depth water to upwell in the east, whereas equatorial divergence drives upwelling along the Equator [19]. In addition to equatorial upwelling, cyclonic gyres such as Guinea Dome in the eastern tropical North Atlantic and Angola Dome in the South Atlantic, along with African coastal upwelling, are regions that facilitate the movement of deeper water masses towards the surface [20]. These waters subsequently flow poleward with Ekman transport, are subducted in the subtropical gyres and close the cell.

The tropics and subtropics of the Atlantic are regions where net evaporation occurs, which increases the salinity of surface waters. In the high tropical latitudes and the central regions of the subtropical oceans, excess evaporation over precipitation produces regional sea surface salinity maxima (SSS_max_), areas of dense high-salinity water [21]. In the North Atlantic, SSS_max_ falls fully within the equatorial-flowing limb of the subtropical gyre in the eastern sector of the subtropical belt. The highest salinities (greater than 37.3) over the entire section are observed in a region of excessive evaporation at the sea surface near 23° N [22]. In the South Atlantic, there is a broad flow towards the northwest, with its SSS_max_ ‘pressed up’ against the western boundary.

After submersion due to Ekman pumping and convection, these waters give rise to salinity maximum water (SMW) [23], often referred to as the subtropical underwater (STUW). This process spreads the high-salinity signature across the entire basin, as the subduction of salty eastern subtropical waters into the thermocline also increases the salinity of western Atlantic thermocline waters [24]. As it progresses equatorward, the STUW can be traced into neighbouring areas as a subsurface maximum in salinity [23], whereas the overlying water is salinity poor because of the high precipitation in the tropics [20]. It is found in the upper 100 m at density ranges of *σ*_θ_ = 25.75 [25] between the oceanic mixed layer and the main thermocline [21].

## 3. Material and methods

The present study is based on planktonic foraminifera specimens from a meridional transect of 16 core-top sediment samples spanning from 31° N to 25° S (figure 1). The sediments consist mainly of foraminiferal marl ooze and correspond to a range of upper water column structures, varying from a strongly stratified, shallow thermocline at the Equator to a much thicker, mixed surface layer at the subtropical gyres. The carbonaceous fossils were found to be exceptionally well preserved, with the exception of samples (8. RC24-10 and 9. RC24-11) from the equatorial upwelling region, where the foraminifera specimens exhibited slight dissolution [5]. All core tops are from the late Holocene in age according to previous studies, with two samples verified by radiocarbon dates for the present study (table 1). Radiocarbon dating was conducted on 10 planktonic foraminifera specimens from each sample via the MICADAS system with a gas interface at the Alfred Wegener Institute in Bremerhaven, Germany [26].

The coarse fraction of each sample was pre-washed with deionized water over a 63 μm mesh and oven dried. Initially, the samples were sieved through a 150 μm sieve, and the planktonic foraminifera fragmentation index was calculated according to Berger *et al*. [27]. The coarse fraction was subsequently sieved into different size categories, with the present analysis focusing on *T. trilobus* specimens from the 300–355 μm fraction. *Trilobatus trilobus* was distinguished from the rest of the *T. sacculifer* plexus morphospecies according to Poole and Wade [28].

The mean weight of the *T. trilobus* specimens from each sample was previously reported in Zarkogiannis *et al*. [5] and was determined by averaging the weights of 50 individual specimens. For this study, these specimens were photographed under a stereomicroscope to document the coiling direction, size and area measurements. Subsequently, 15 specimens from each sample were analysed via μCT for biometry and preservation assessment. These specimens provided sufficient mass (at least 350 μg of carbonate) for subsequent geochemical analyses, including trace element and stable isotope determination. In line with standard practices in the relevant literature, which typically recommend approximately 350 μg of material for geochemical analyses [29], 50 specimens for weighing [30] and eight specimens for μCT scanning [31], the number of specimens used in each analysis ensures reliable results between methods.

After determining the different shell traits from the physical analyses and the environmental variables from the atlases, I used standard least squares linear regression analysis to examine the relationships between the traits and the environment.

### 3.1. Stereomicroscopic analysis

From each sample, approximately 50 pre-weighed samples [5] were fixed, with their umbilical side facing upwards, to micropalaeontological slides via tragacanth glue. The analysis enabled the calculation of the foraminiferal area density (*ρ*_A_; µg µm^−2^) [32], which is a normalization method used to account for changes in foraminiferal shell mass due to specimens’ size variations. Additionally, it facilitated the determination of the prevailing coiling direction of the specimens in this region.

Images were acquired via a modular Leica M165 C fully apochromatic stereomicroscope with an integrated 10 megapixel Leica IC90 E colour camera (at 2× magnification) and processed via ImageJ software (version 1.50i). The image analysis system automatically determines shell two-dimensional (silhouette) areas and Feret diameters for each batch. Instead of using Feret’s diameter, which measures the longest distance between only two points along the selection boundary, the ‘equivalent circular diameter’ or nominal diameter was used. This metric represents the diameter of a circle with the same area as the silhouette area of the specimen, making it a more accurate metric for denoting the average length of each specimen. Calibration for the silhouette area measurements was performed via a microscale images taken at the same magnification as the foraminiferal images (2×). The foraminiferal area density was determined by dividing the average shell weight by the corresponding mean silhouette area.

### 3.2. X-ray micro-computed tomography

To study the test biometry and assess their preservation state, on average 15 *T*. *trilobus* shells from each sample were scanned via a GE/Phoenix v|tome|x s 240 CT scanner at the Geozentrum (Nordbayern). The tests were fixed in a customized cylindrical container together with a calcite microcrystal [33] that was used to standardize the CT number (grey levels) of the scanned specimens. The CT number, which is an indicator of calcite density, is the normalized value of the calculated X-ray attenuation coefficient of a voxel in a computed tomogram. In this study, calcite standard crystal and foraminiferal test samples were measured simultaneously to standardize the CT number for each specimen. The CT number is defined as the linear transformation of the original linear attenuation coefficient of voxels [34]. A high-resolution setting (voltage of 80 kV, current 80 μA, detector array size of 1024 × 1024, 1501 projections/360°, 2.5 s/projection) enabled the acquisition of three-dimensional (3D) images with an isotropic pixel size of approximately 1.2 μm. Phoenix datos|x 2.0 software, which uses the general principle of the Feldkamp cone beam algorithm to reconstruct image cross-sections from filtered back projections, was used to correct and reconstruct tomographic data.

The dissolution of the specimens was assessed on the basis of their CT numbers following the protocol of Iwasaki *et al*. [34]. Segmentation was performed in Avizo^®^ software, and the CT number, test thickness, volume and surface area were calculated. In this study, the test thickness was calculated by dividing the test volume (μm³) by the test surface area (μm²) [33]. This ratio of test volume to test surface area serves as a reliable approximation of the average test thickness [35]. The extent of contamination in the fossil shells was determined by separately segmenting the sedimentary impurities filling the shell, and the debris volume was calculated as a percentage of the total shell volume. Furthermore, by using the ambient occlusion algorithm in Avizo^®^ software the ‘potential specimen volume’, i.e. the total volume if the cell was alive, and the chambers were filled with protoplasm (i.e. biovolume + shell), and ‘potential’ outer surface area were approximated. This allowed the determination of ‘potential’ cell surface area to volume ratios (SA:V) and bulk shell densities (BSDs).

### 3.3. *In situ* oceanographic data

The mean annual and monthly ocean temperature and salinity data from the period between January 2004 and January 2020 were extracted for each core site from the International Argo Ocean Monitoring programme [36]. These temperature data were utilized to determine *T. trilobus* ACDs at each location, and the salinity data at these depths (salinity ACD_Mg/Ca_) were extracted for comparison with the various shell traits. The Argo data, which are quality controlled, represent the first 300 m of the water column. Instead of extrapolating single-point hydrographic data at the exact core coordinates, the surface (2.5 m) temperature and salinity values of each site were extracted from a grid area of 0.1 × 0.1 decimal degrees (approx. 10 × 10 km) around the site location (all data are given in electronic supplementary material, table S1).

Within the first 300 m, hydrographic parameters were provided for 25 depth points, starting from 2.5 m and then at intervals of every 10 m up to a depth of 200 m, every 20 m up to 300 m. For most of the cores, annually averaged temperature and salinity data from the overlying water column were used except from the three northernmost (1. VM27-261, 2. VM19-308 and 3. VM16-206) and the three southernmost cores (14. VM16-36, 15. RC08-19 and 16. RC08-23) from the subtropical gyres. For these cores, the oceanographic parameters were averaged only for the three warmest months, since studies have shown that the largest flux of *T. sacculifer* occurs during the warmest months of the year at temperate latitudes [6,37]. For the Northern Hemisphere sites, the highest temperatures were usually recorded from August to October, whereas for the southern sites, the highest temperatures were recorded from February to April.

Ocean carbonate system data were taken from GLODAP v. 1.1 [38]. Given the late Holocene age of the core tops, the modern seawater data are corrected for the acidifying influence of anthropogenic CO_2_, by subtracting the GLODAPv1.1 anthropogenic dissolved inorganic carbon (DIC) estimates from the total DIC values to provide an estimate of pre-industrial DIC. These values, along with paired alkalinity, nutrient and hydrographic data, were used to make pre-industrial CO_2_ system determinations for GLODAP via CO2SYS v. 1.1 [39]. This dataset was then imported to Ocean Data View [40], and carbonate ion concentrations were obtained for each site at a range of depths via Ocean Data View’s 3D estimation tool. For consistency, further oceanographic parameters, such as 3D salinity and 3D density, were calculated for the same depth ranges via the same software.

### 3.4. Estimation of calcification depths and temperatures

Apparent calcification depths were determined via shell elemental geochemistry. Specifically, 15 μCT-scanned specimens (approx. 350 μg of foraminiferal carbonate) from each sample were subsequently utilized for trace element determination (Mg/Ca), following the method outlined by Barker *et al*. [41]. The treatment and analysis were performed in the metal-free suite of the Department of Earth Sciences, University of Oxford (UK) using a Perkin Elmer NexION 350D inductively coupled plasma mass spectrometer (ICP-MS). The instrumental precision of the ICP-MS was monitored every five samples via analysis of an in-house standard solution with a Mg/Ca of 2.93 mmol mol^−1^ (long-term standard deviation of 0.026 mmol mol^−1^ or 0.88%). Given the small analytical uncertainty, the temperature uncertainty is influenced primarily by the uncertainties of the calibration equation. The ECRM752‐1 limestone standard, with a reported Mg/Ca of 3.75 mmol mol^−1^, was analysed (*n* = 3) to allow interlaboratory comparisons [42], with an average of 3.85 ± 0.027 mmol mol^−1^. To monitor the cleaning efficacy, Al/Ca, Fe/Ca and Mn/Ca were measured alongside Mg/Ca. None of these ratios showed covariance with Mg/Ca (electronic supplementary material, figure S1). Duplicate analyses were performed at three sites, specifically sites 8 (RC24-10), 14 (VM16-36) and 16 (RC08-23), and all were found to span the temperature range of the study. The propagated uncertainty, accounting for the analytical precision and the standard deviation of each of the two replicates when averaged, yielded a value of 0.14 mmol mol^−1^. This resulted in an average error of 0.8°C during the temperature conversion using the calibration equation provided below.

Shell Mg/Ca was converted to seawater temperatures using the Regenberg *et al*. [43] calibration equation for *T. trilobus* (Mg/Ca = 0.6 (± 0.16) × exp (0.075 (± 0.006) × T°C)), which was derived for the same area and produced reasonable temperatures along the entire transect compared with other equations (electronic supplementary material, figure S2). The Mg/Ca reconstructed temperatures (T_Mg/Ca_) for each sample were compared with the mean annual temperature Argo profile for sites between 23° N and 23° S and the mean temperatures of the three warmest months of the year north and south of 23° latitude in accordance with Hertzberg and Schmidt [44]. The water depth where shell T_Mg/Ca_ matches that of the *in situ* water temperature profile is considered to reflect the calcification depth of the respective species at the site.

## 4. Results

### 4.1. Calcification depths and *in situ* oceanographic data

**Figure 2.**
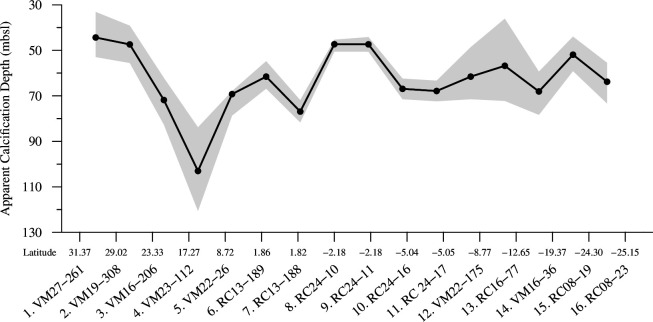
Apparent calcification depth (ACD) estimates of *T. trilobus* at metres below sea level (mbsl) based on bulk shell Mg/Ca ratio. The shaded areas depict the 1σ confidence interval.

The proposed apparent calcification depths (ACD_Mg/Ca_) are based on Mg/Ca temperature estimates. Along the transect, the studied planktonic foraminifera species appear to inhabit the surface water layers. Despite significant variations in estimated calcification depths between most of the sites, depth values in the South Atlantic between the gyres and the tropics converge to an average of approximately 63 m. The shallowest habitats at approximately 45 m are estimated for the northernmost samples from the gyre, whereas sample 4 (VM23-112) appears as an outlier with a reconstructed depth value of 103 m (figure 2). There is a clear indication of habitat shoaling at southern equatorial sites 8 (RC24-10) and 9 (RC24-11), where depths as shallow as approximately 47 m are estimated. The uncertainty in the geochemically inferred calcification depths averaged 8.3 m, with variations of up to approximately 50%, depending on the thermal structure variability of the water column at each sampling site.

Based on the Mg/Ca derived calcification depth estimates from the Argo data, additional physical and chemical oceanographic parameters, such as (pre-industrial) carbonate ion concentrations, salinity, *in situ* density and *σ*_*θ*_ (potential density), were extrapolated and calculated at these ACDs. *σ*_*θ*_ reveals the isopycnal layers or water column strata and can occupy different depths. The amplitudes of the corresponding *σ*_*θ*_ values to the reconstructed depth of each species for all the sites are summarized in table 2. *Trilobatus trilobus* is found within a fairly restricted isopycnal horizon (Δ*σ*_*θ*_ = 0.8). Close to the Equator, it is reconstructed to inhabit lighter water layers of *σ*_*θ*_ = 24.5, whereas in the subtropics, it is found up to the *σ*_*θ*_ = 25.3 isopycnal.

**Table 2.** List of the studied samples and their locations together with the results of the weighing and geochemical analyses and the *in situ* physicochemical oceanographic parameters at ACD_Mg/Ca_ (see §3.3 for details). Shell weights are from Zarkogiannis *et al*. [5]. Mg/Ca was converted to temperatures using the Regenberg *et al*. [43] equation (see §3.4 for details).

sample	shell weight (μg)	Mg/Ca (mmol mol^−1^)	temperature (°C; Mg/Ca)	*T. trilobus* ACD_Mg/Ca_ (m)	salinity ACD_Mg/Ca_	density (σθ)	CO_3_^2−^ (3D; μmol kg^−1^)	salinity (3D)	water density (3D; *σ*_*θ*_)
1. VM27-261	31.2	3.62	24.0	44.3	36.9	25.12	250.3	36.6	26.0
2. VM19-308	30.2	3.80	24.6	47.4	37.1	25.08	285.4	37.0	25.9
3. VM16-206	32.2	4.00	25.3	71.8	37.4	25.06	289.4	37.3	25.5
4. VM23-112	32	3.64	24.0	103.0	37.3	25.34	269.2	36.9	25.0
5. VM22-26	28.3	3.50	23.5	69.2	36.6	24.71	285.7	36.1	24.8
6. RC13-189	27.9	3.99	25.3	61.6	36.0	24.76	250.1	36.0	24.3
7. RC13-188	27.3	3.57	23.8	76.9	36.0	24.50	231.1	36.0	24.8
8. RC24-10	24.1	2.92 (2.80)	21.1	47.3	35.8	25.10	235.8	35.9	24.2
9. RC24-11	22.1	2.92	21.1	47.4	35.8	25.10	235.8	35.9	24.2
10. RC24-16	26.7	3.15	22.1	66.9	36.0	24.96	251.2	35.8	24.6
11. RC24-17	25.6	3.19	22.3	67.9	36.0	24.63	250.4	35.8	24.6
12. VM22-175	27.8	3.82	24.7	61.6	36.4	24.51	254.6	36.5	24.4
13. RC16-77	28.8	3.53	23.6	56.8	36.8	25.11	259.2	36.7	24.9
14. VM16-36	29.8	3.25 (3.24)	22.5	68.1	36.5	25.18	260.2	36.5	24.9
15. RC08-19	29.2	3.51	23.5	51.9	36.6	24.96	258.4	36.4	24.9
16. RC08-23	31.6	3.09 (3.59)	21.8	63.8	36.3	25.29	252.2	36.1	25.1

### 4.2. *Trilobatus trilobus* shell weights

**Figure 3.**
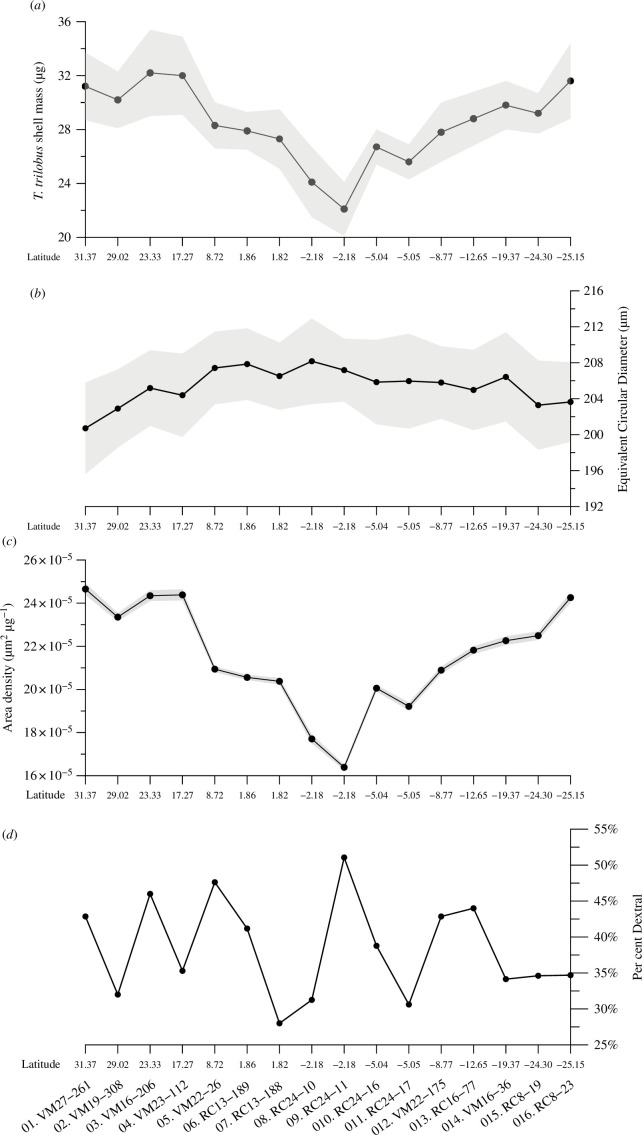
Along the studied transect (*a*) mean *T. trilobus* shell masses from Zarkogiannis *et al*. [5], (*b*) equivalent circular diameter as an average size indicator, (*c*) area density *ρ*_A_ or silhouette normalized weight and (*d*) coiling direction of *T. trilobus* shells showing the percentage of dextral specimens in the population. The shaded areas depict the 1σ confidence interval.

The investigated size fraction (300–355 μm) of *T. trilobus* specimens was reported in Zarkogiannis *et al*. [5] to be lighter at equatorial sites and heavier in the subtropics (figure 3*a*) following *in situ* salinities. In the present study, compared with the *in situ* oceanographic variables (table 3), shell weights were also found to depend mostly on salinities (linear regression *r*^2^ = 0.69, *p* < 0.01) and, to a lesser extent (*r*^2^ = 0.39, *p* < 0.05), on CO_3_^2−^ concentrations ([CO_3_^2^]). If the two (8 and 9) partially dissolved samples are omitted, the shell weights strongly correlate with the ambient seawater (3D) density (*r*^2^ = 0.61, *p* < 0.01).

**Table 3.** Table summarizing the coefficient of determination (*r*^2^) of the linear regression analyses. Values in italics denote the degree of anti-correlation between the variables. Values in bold represent the highest coefficient of determination for each comparison.

	shell weight	bulk shell density	shell thickness	shell percent	*ρ*_A_	SA:V	shell volume	silhouette area
Salinity at ACD_Mg/Ca_	**0.69**	**0.35**	**0.78**	**0.80**	**0.70**	*0.19*	0.60	*0.38*
(3D) CO_3_^2−^	0.39	0.08	0.63	0.49	0.35	*0.27*	0.63	*0.07*
(3D) salinity	0.55	0.33	0.71	0.70	0.55	*0.08*	0.46	*0.29*
(3D) density (σ_θ_)	0.58	0.22	0.72	0.70	0.67	***0.28***	**0.66**	***0.70***

### 4.3. Light microscopy and photographic analyses

The 300–355 μm sieve fraction allows a maximum of approximately 18% variation in test size. Within the sieve fraction, specimens from the tropical and equatorial sites were found to be larger than those from the subtropical gyres. Compared with that of the subtropical gyres, the specimens’ two-dimensional silhouette area was found to be approximately 7% larger at the tropical and equatorial sites (table 4), and their equivalent circular diameter approximately 4% larger (figure 3*b*). The variability in size is greater in the Northern Hemisphere, with the smallest specimens within the northern subtropical gyre and the largest at the northern equatorial sites (south of 10° N), after which the number of samples gradually decreases towards the southern subtropical gyre. The silhouette area showed a significant anti-correlation with ambient water densities (*r*^2^ = 0.7, *p* < 0.01).

**Table 4.** Light microscope and μCT spatial information of fossil *T. trilobus* shells across the central Atlantic. The biovolume is the hollow space within the fossil foraminifera shell.

site	silhouette area (mm²)	potential (specimen) volume (nl)	calcite (test) volume (nl)	biovolume (nl)	outer surface area (mm²)	debris (%)
1. VM27-261	0.126 ± 13%	20.807 ± 13%	9.705 ± 19%	9.988 ± 11%	0.436 ± 10%	12
2. VM19-308	0.129 ± 11%	22.374 ± 12%	11.231 ± 9%	12.185 ± 18%	0.519 ± 8%	7
3. VM16-206	0.132 ± 10%	21.689 ± 8%	9.991 ± 13%	11.698 ± 15%	0.510 ± 6%	7
4. VM23-112	0.131 ± 11%	21.291 ± 13%	9.384 ± 8%	11.907 ± 19%	0.497 ± 11%	5
5. VM22-26	0.135 ± 10%	22.429 ± 12%	9.259 ± 20%	13.630 ± 16%	0.512 ± 8%	7
6. RC13-189	0.136 ± 10%	20.170 ± 11%	7.617 ± 24%	12.554 ± 14%	0.488 ± 8%	7
7. RC13-188	0.134 ± 9%	20.482 ± 13%	7.132 ± 24%	13.350 ± 12%	0.487 ± 9%	8
8. RC24-10	0.136 + 8%	20.480 ± 14%	6.653 ± 21%	13.827 ± 15%	0.549 ± 11%	3
9. RC24-11	0.135 ± 11%	19.264 ± 13%	6.352 ± 18%	12.912 ± 18%	0.467 ± 9%	4
10. RC24-16	0.133 ± 11%	20.612 ± 12%	7.385 ± 21%	13.227 ± 15%	0.496 ± 9%	2
11. RC24-17	0.133 ± 13%	21.786 ± 7%	8.836 ± 15%	13.550 ± 13%	0.509 ± 5%	6
12. V22-175	0.133 ± 10%	19.399 ± 10%	8.113 ± 15%	11.286 ± 14%	0.475 ± 7%	2
13. RC16-77	0.132 ± 11%	18.205 ± 11%	7.418 ± 12%	10.786 ± 17%	0.452 ± 9%	5
14. VM16-36	0.134 ± 12%	20.595 ± 13%	8.520 ± 16%	12.075 ± 15%	0.506 ± 9%	9
15. RC8-19	0.130 ± 12%	17.584 ± 16%	7.347 ± 18%	10.237 ± 20%	0.452 ± 10%	3
16. RC8-23	0.130 ± 11%	19.822 ± 12%	8.459 ± 13%	11.363 ± 16%	0.477 ± 8%	10

After dividing average shell weights by their corresponding mean silhouette area, the foraminiferal area density (*ρ*_A_; µg µm^−2^) is calculated, which is a normalization method used to account for changes in foraminiferal shell mass due to specimen size variations [32]. Since the specimens are size restricted, the area density *ρ*_A_ is predominantly controlled by changes in the shell mass. It thus follows the weight record by being higher in the subtropics and approximately 33% lighter at the equatorial sites (figure 3*c*). *ρ*_A_ was found to correlate very strongly with *in situ* salinities (*r*^2^ = 0.7, *p* < 0.01) and only moderately (*r*^2^ = 0.35, *p* < 0.05) with [CO_3_^2–^]. If again the two partially dissolved samples 8 (RC24-10) and 9 (RC24-11) are omitted, *ρ*_A_ correlates more strongly (*r*^2^ = 0.63, *p* < 0.01) with ambient densities, whereas the correlation with [CO_3_^2−^] becomes insignificant (electronic supplementary material, table S2).

Coiling directions were measured in 765 specimens from the *T. trilobus* morphospecies (figure 3*d*). When all the samples are grouped together as a whole, the modern *T. trilobus* population from the central Atlantic Ocean shows 39% dextral (61% sinistral) coiling. The individual sample averages are variable (28–51%), all favouring sinistral coiling, except for one.

### 4.4. μCT analyses

**Figure 4.**
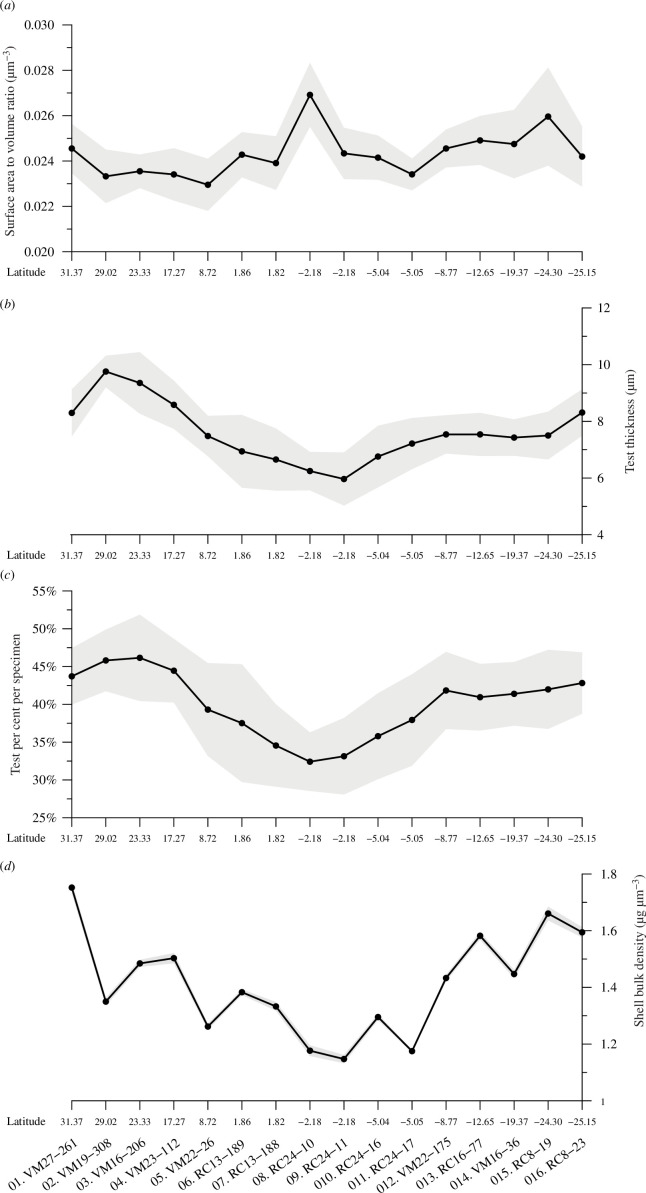
*Trilobatus trilobus* biometric test properties along the transect from μCT analyses: (*a*) potential surface area to volume ratio (SA:V), (*b*) average test thickness or ‘specific surface area’, (*c*) test volume as a percentage of the total potential volume and (*d*) bulk shell density or ‘volume normalized shell weight’. The shaded areas depict the 1σ confidence interval.

The segmentation and subsequent spatial analysis of the high-resolution CT images revealed some novel biometric information of the individual foraminifera (as inferred from their shells), which is rare in foraminifera studies. The major results are summarized in figure 4 and table 4. *Trilobatus trilobus* specimens from the 300–355 μm sieve fraction occupy, on average, a volume of 20 000 000 μm^3^ when filled completely with protoplasm and lacking spines. The largest specimens (of up to approx. 22 × 10^6^ μm^3^) are found within the core of the northern subtropical gyre southern region, where *T. trilobus* shells (gradually) decrease in size to approximately 23% to become about 17.5 × 10^6^ μm^3^ in the southernmost samples (table 4). Among all the different *in situ* and reconstructed variables tested, the *T. trilobus* potential volume was weakly dependent (*r*^2^ = 0.24, *p* < 0.1) on [CO_3_^2−^] and the calcification depth ACD_Mg/Ca_ (*r*^2^ = 0.2, *p* < 0.1). However, the *T. trilobus* test volume (i.e. calcite volume; table 4) was strongly correlated with ocean density (*r*^2^ = 0.66, *p* < 0.01) and [CO_3_^2−^] (*r*^2^ = 0.63, *p* < 0.01). Furthermore, the shell weight is correlated with the test volume (*r*^2^ = 0.55, *p* < 0.01) but not with the overall (potential) specimen volume, which also does not correlate with the silhouette area.

The virtual filling by segmentation of the chambers and foramina (openings in the shell walls) allowed the determination of the outer surface area to volume ratio (SA:V) of 216 *T. trilobus* tests, which is on average 0.024 μm^−1^ in the central Atlantic. SA:V is a sphericity index since the surface area decreases for rounder shapes; thus, lower ratios characterize more spherical objects. The SA:V gradually increased from the northern to southern sites (figure 4*a*) by approximately 13%, since the SA:V increased with decreasing volume. The surface area-to-volume ratios were somewhat inversely correlated with ocean density (*r*^2^ = 0.28, *p* < 0.05) and [CO_3_^2−^] (*r*^2^ = 0.26, *p* < 0.05), implying that in lighter, carbonate ion-depleted waters, *T. trilobus* specimens become smaller and increase in SA:V ratio. With an average of approximately 500 000 μm^2^, the specimens’ potential outer surface area exhibited the least intra- (9%) and inter- (12%) sample variability (table 4). This makes the potential outer surface area the most conservative cell property, and it did not correlate with any of the oceanographic variables considered here.

The ratio of test volume/test area (‘specific surface area’) provides a linear dimension (μm) that can be used as a measure of average test thickness. On average, *T. trilobus* tests are relatively thick in the subtropics and become thinner by almost 40% at equatorial sites (figure 4*b*). The thickest *T. trilobus* tests, with an average thickness of 9.8 μm, are found in sample 2 (VM19-308) from the northern subtropical gyre. Test thickness strongly correlates with shell weight (*r*^2^ = 0.72, *p* < 0.01) since the ‘specific surface area’ should essentially be an indication of how massive an object is. The thinnest specimens (6 μm) are found just south of the Equator. For most of the South Atlantic sites, the test thicknesses are relatively stable at approximately 7.5 μm. Thickness also showed a very strong correlation with salinity (*r*^2^ = 0.81, *p* < 0.01) and, to a lesser extent (*r*^2^ = 0.63, *p* < 0.01), with [CO_3_^2−^].

The average volume that the calcareous test occupied within the foraminifera individual was also calculated as a percentage of the average potential volume (figure 4*c*). The hard tests occupy on average 40% of the total *T. trilobus* specimen and the remaining 60% could potentially be occupied by organic material in living specimens. These proportions change with latitude, and in North Atlantic subtropical regions, individuals consist of approximately 45% carbonate material, whereas at the Equator, the percentage decreases by up to 30%, and the test may account for only approximately 1/3 (32%) of the total specimen. In South Atlantic subtropical regions, the test occupies about 40% of the foraminiferal specimen and this percentage is less variable. The test percentage strongly depends on the *in situ* seawater salinity (*r*^2^ = 0.80, *p* < 0.01). Overall, in the subtropical regions, *T. trilobus* accommodates/sequests (by volume) only slightly more organic than inorganic carbon does, whereas in the equatorial regions, the organic part of the sample is double that of the inorganic part (table 4).

To account for changes in average shell weights (figure 3*a*), owing to specimen size variability within the sieved fraction, for each sample, the average shell weights were normalized to the average potential specimen volumes yielding BSDs (figure 4*d*). The mean BDS of *T. trilobus* is 1.4 g cm^−3^ and has an average ACD of 60 m (sea water density of 1.025 g cm^−3^. Based on this, the organic tissue of *T. trilobus* is estimated to have an average density of 0.375 g cm^−3^ (table 4). BSDs, as indicators of calcification effort, follow the same pattern as shell weights and test percentages, with higher values in the subtropics and a reduction of up to 35% in the (southern) equatorial regions. However, in contrast to the other two measures, it appears that the skeletal density of *T. trilobus* is greater in the South Atlantic because of the decrease in potential specimen volume (the BSDs of sample 1 (VM27-261) may, to some extent, be artificially elevated due to sedimentary debris contamination (table 4)). BSDs are best correlated with *in situ* salinities (*r*^2^ = 0.35, *p* < 0.05), but if the two partially dissolved (see §5) samples 8 (RC24-10) and 9 (RC24-11) are omitted, the correlation with salinity becomes insignificant, and the BSD correlates strongly with *in situ* water density (*r*^2^ = 0.35, *p* < 0.05).

**Figure 5.**
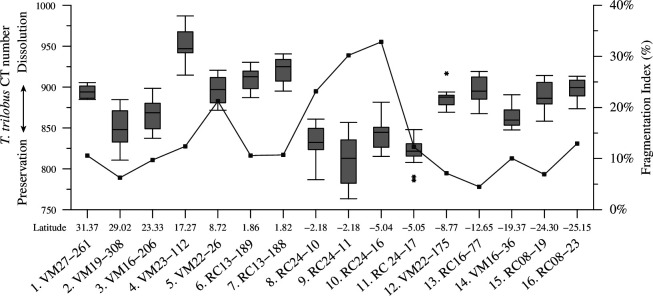
Box-and-whisker plots of *T. trilobus* test mean CT number (test relative densities) specimens with latitude. Boxes extend from the lower to upper quartile values of the data, with a line at the median. Whiskers indicate 1.5 times the interquartile distance. Asterisks are outliers. The dashed line represents the superimposed planktonic foraminifera fragmentation index from Zarkogiannis *et al*. [5].

In addition to providing information about shell biometry, tomographic analyses also allow assessment of the integrity of the tests, i.e. their preservation state. With the exception of the two equatorial samples 8 (RC24-10) and 9 (RC24-11), *T. trilobus* specimens were found well preserved in the central Atlantic (figure 5). The CT number is an indicator of a test’s relative density, and the higher the number is, the higher the test’s integrity [34,45]. The samples from the northern extra-equatorial sites, with a CT number of approximately 900, were the best preserved, whereas dissolution was highest in the southern equatorial locations (CT number approx. 800). Test preservation below the two subtropical gyres was comparable. Interestingly, the shell CT numbers were strongly correlated with BSD (*r*^2^ = 0.7, *p* < 0.01), which was far greater than the correlation with the sieved-based weights (*r*^2^ = 0.42, *p* < 0.01). Although the foraminifera fragmentation index (FI) is high below the equatorial regions, overall it does not correlate significantly with CT number (*r*^2^ = 0.26, *p* < 0.1). In contrast, FI is related to the percentage of skeletal mass in the foraminifer shells (test %; *r*^2^ = 0.45, *p* < 0.01). These results support the idea that rather than being an actual dissolution indicator, the FI refers mostly to the rigidity of the foraminiferal tests.

## 5. Discussion

The present analysis of reflected light microscope images, combined with high-resolution images from the μCT acquisition, provides valuable information regarding the biometry and shell integrity of foraminifera in central Atlantic surface sediments. The specimens were well preserved, and within the studied restricted size fraction, *T. trilobus* shells were thinner and slightly larger at the Equator, which is consistent with findings for other species in the area, such as *Globigerinoides ruber* and *Globorotalia truncatulinoides* [5]. Their coiling direction was predominantly sinistral in this region, comparable to what is known from modern populations in other areas [28,46].

The carbonate content of *T. trilobus* was found to be primarily a function of seawater salinity. According to the results above, *in situ* salinity was found to be correlated with the average (sieved-based) shell weight, area density (*ρ*_A_; size normalized weight), test thickness and test percentage within the foraminifera individual (table 3). Shell weights and BSDs were also found to depend on *in situ* water density but to a lesser extent on [CO_3_^2−^]. Calcium carbonate precipitation depends on the concentrations of calcium and carbonate ions in the water. Even with low carbonate ion concentrations, a solution may favour CaCO_3_ precipitation if the concentration of calcium is high [47]. The surface concentration of calcium in this region is dependent primarily on salinity because the salinity represents the concentration of total solids in solution. Consequently, any changes in salinity due to variations in evaporation or precipitation affect the concentrations of all salts, including calcium, similarly. Moreover, independent of the quantity of calcium or carbonate, carbonate precipitation may also be favoured because of the effect of salinity or the concentration of ions other than calcium on the solubility product [48].

Previous culturing studies revealed a strong dependency of *T. sacculifer* plexus shell weight on carbonate ion concentration [49] and/or pH [50]. However, in these studies the carbonate system was manipulated with acid titration, whereas in the natural system the pH may also change as a function of DIC concentration, creating different conditions. In the surface central Atlantic, changes in DIC are indeed related to both the temperature and salinity (i.e. density) of the waters [51]. Despite locally lower DIC concentrations, *T. trilobus* at the Equator is able to accommodate higher biovolumes (table 4) and thus does not appear to be constrained by carbon availability. Since foraminifera are able to internally control the calcite saturation state [52,53], changes in the degree of inorganic carbon precipitation for shell formation may be driven by physical oceanographic conditions for buoyancy and depth habitat adjustment. This may explain the strong correlations of the different shell calcifications considered here with the salinity and density of the ambient seawaters.

Salinity impacts the incorporation of a variety of additional elements in the calcite lattice such as Mg^2+^, Sr^2+^, Na^+^, Li^+^, etc. [54–58], either directly or indirectly by affecting calcification rates [59]. The repercussions of these mineralogical modifications on test density, and consequently, its overall mass, remain unknown. Foraminifera may incorporate within the CaCO_3_ lattice different solids, potentially as a means of modulating the density of their biomineral, i.e. the total test mass, for density matching and buoyancy regulation. The extent of alteration in the biomineral’s density is contingent upon the degree to which calcium ions (Ca^2+^) are replaced by elements with higher (Sr^2+^) or lower atomic mass (Mg^2+^). Although bivalent Mg^2+^ ions may substitute Ca^2+^ in the crystal lattice [60], a salinity-induced increase in monovalent ions [58,61] that occupy interstitial positions in the calcite structure [62] may have an overall effect on test’s mass. The idea of foraminifera tests produced by biominerals of slightly different densities is further supported by the strong correlation between the CT number and BSD, which in turn showed some correlation with *in situ* water density.

The contamination of shell weight measurements by sedimentary infillings as a source of uncertainty is probably minimal, given that the volume of impurities (debris %; table 4) is relatively low and remains stable along the transect. Furthermore, there was no observed correlation between the weight measurements and the volume of such impurities. Although traits involving shell weight measurements may be subject to contamination, traits derived from μCT analyses, such as test thickness or volume, may be more precise indicators of calcification. The significant correlation found between test volume and ocean density supports the hypothesis that the foraminifera shell plays a role in buoyancy regulation [63,64]. Moreover, the foraminifera shell does not need to be fully filled to function as ballast, as foraminifera have been observed to only partially fill their last chamber. Retraction of the lighter organic protoplasm to the inner chambers changes the organism’s density/weight, causing the empty chambers to act as a sinker. In *T. trilobus*, since the biovolume exceeds the test volume, ample space is available for buoyancy regulation. However, the smaller variability in biovolume (13%) compared with the test volume (17%) in the studied specimens may suggest that maintaining certain levels of organic tissue is necessary, and protoplasm retraction might be a stress response perhaps for rapid displacement into deeper waters.

**Figure 6.**
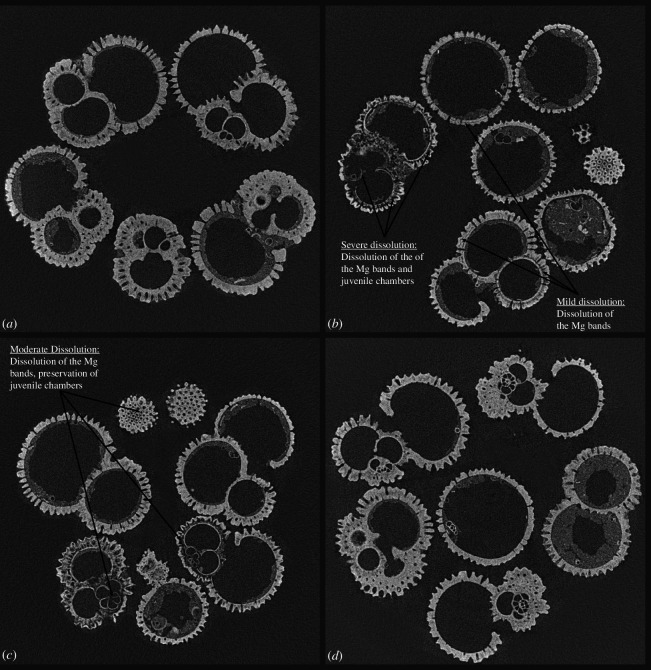
X-ray tomographs of four of the studied samples; (*a*) bright, thick and well-preserved specimens from site 4 (VM23-112) in the North Atlantic; (*b*) relatively bright, thin and somewhat dissolved overall specimens from site 9 (RC24-11), some of which have lost juvenile chambers; (*c*) relatively bright, thin and moderately dissolved overall specimens from site 8 (RC24-10) that have lost some Mg^2+^-rich calcite but retain the juvenile chambers; (*d*) bright, thick and well-preserved specimens from site 13 (RC16-77) in the South Atlantic.

With respect to calcite dissolution, the examined tests were generally well preserved, especially in subtropical Atlantic regions (figure 6*a,d*). However, carbonate shells deposited beneath the south equatorial upwelling areas exhibited slight dissolution. Some specimens, particularly in samples 8 (RC24-10) and 9 (RC24-11), were significantly impacted by dissolution, probably due to (i) the loss of the most susceptible higher magnesium shell wall bands [65] and/or (ii) juvenile chambers [66] (figure 6*b,c*). These findings are consistent with the μCT analysis of the shells of two additional species from the same samples [5]. The Mg/Ca values of the present study agree with previous analyses on *T. trilobus* [15,67]. As suggested by figure 2, these values denote temperature changes in the depth habitat of these organisms rather than dissolution, since severe dissolution would have spuriously decreased Mg/Ca in the shells, which would have indicated artificially deeper habitats at the Equator.

The process of dissolution initiates internal corrosion of the chamber walls or septa within the foraminifera shells (figure 6*a,d*). This dissolution phenomenon is notably associated with the well-documented Mg-banded lamellar calcite layering, as observed in prior research [65]. While the partial dissolution of these shells indeed exerts an influence on metrics such as weight measurements, as well as the subsequent area and volume normalization procedures, it does not always distort test geometrical information. It is noteworthy, for instance, that the gradual advancement of dissolution-induced effects does not engender any alterations in fundamental shell dimensions, such as thickness and overall size [68]. Therefore, it is plausible to infer that shell volumetric and topological information (total and test volume, surface areas, SA:V), as discerned through tomographic analyses, holds a heightened degree of reliability when investigating the degree of foraminifera calcification by means of their fossilized shells.

According to the geochemically derived ACD_Mg/Ca_ values, which falls within the published estimates for the area [69,70], *T. trilobus* exhibit a shoaling trend in the habitat depth towards southeastern equatorial sites (figure 2). This shoaling trend aligns with the shoaling of the thermocline at these sites, which is a result of the known west–east slope in the upwelling region in the equatorial Atlantic. This slope causes the thermocline to be shallower in the eastern equatorial Atlantic compared with the western tropical Atlantic [69,71]. A similar shoaling trend towards the Equator is also observed in the water column’s salinity maximum horizon, denoted as the turning points in the T–S curves of each site (figure 3 in [5]). This pattern can be attributed to the accumulation and subduction of waters occurring at the centre of gyres, leading to distinct salinity profiles within the water masses. In these regions, salinities are highest at the surface and gradually decline with depth without experiencing salinity inversions [72].

**Figure 7.**
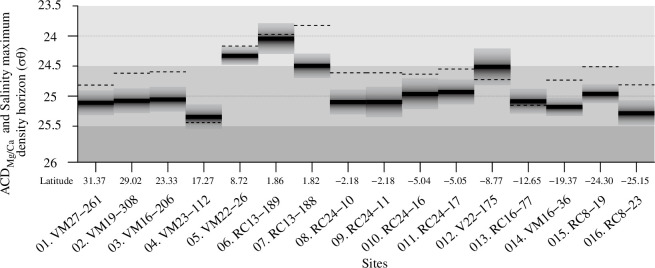
Subsurface salinity maximum (dashed lines) plotted together with the apparent calcifying density horizons reconstructed from the geochemical signal by matching reconstructed Mg/Ca temperatures with *in situ* Argo oceanographic data. The shaded areas depict the 1σ confidence interval.

The density horizons where each site’s salinity maximum is located are plotted together with the seawater density at the ACD_Mg/Ca_ of the studied species in figure 7 (dashed lines). The calcification density horizons exhibit close clustering between 50 and 100 m water depths and correlate significantly (*r*^2^ = 0.6, *p* < 0.01) with the density horizon in which the salinity maximum waters lie. These findings align with prior research emphasizing the importance of density in determining the depth of the habitat of planktonic foraminifera [63,70,73]. If the gyre water masses of no subsurface salinity maximum are considered (figure 7; dotted lines), the coefficient decreases slightly (*r*^2^ = 0.53), but the correlation still remains significant (*p* < 0.01). Figure 7 suggests that *T. trilobus* in the central Atlantic typically inhabits a density layer just below the salinity maximum (density) horizon, which is in line with observations from the equatorial Indian [74] and Pacific Oceans [75], where *T. trilobus* calcification depth was also related to the subsurface salinity maximum zone.

**Figure 8.**
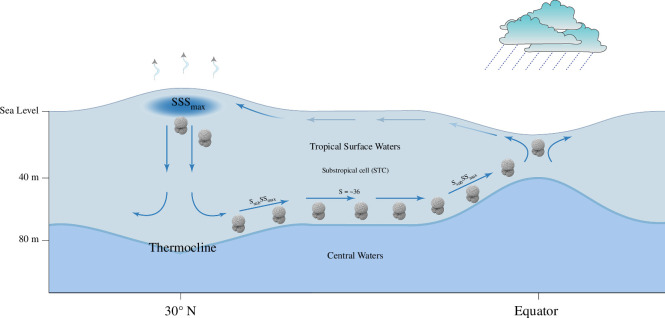
Conceptual model of changing *T. trilobus* habitat within the northern subtropical Atlantic circulation cell. The blue arrows depict the circulation within the subtropical cells (STCs). The same geometry is also hypothesized for the southern subtropical cell.

In both hemispheres, subducted salinity maximum waters flow towards the tropics within the pycnocline confined in the STCs [17]. The Atlantic STCs are shallow overturning circulation systems in the upper 500 m that connect the subtropical subduction zones with upwelling zones in the tropics [76], providing habitat for the studied foraminifera species since they were found to calcify slightly below the salinity maximum density horizon. A conceptual model of the foraminifera habitat within the STC is presented in figure 8. The subsurface STC branches carry thermocline water to the Equator either in western boundary currents (after circulating across the basin in the subtropical gyres) or directly in the ocean interior. The thermocline flows supply eastwards undercurrents that upwell along the Equator or at the eastern boundary. The STCs are driven poleward by surface currents (largely Ekman transport), which return the upwelled waters to the subtropics (e.g. [77]).

Even though most planktonic foraminifera have a large temperature tolerance of about 14–32°C [78,79], they all have an individual, far more restricted optimum temperature (e.g. 23.5°C for *T. trilobus*) at which chamber formation, gametogenesis and food acceptance are highest [78]. In contrast, the salinity tolerance range in planktonic species is wider than the variations encountered in the open ocean (e.g. 24–47 in *T. trilobus*), and although it may play a marginal role in the general foraminiferal distribution, in line with previous studies [74], it was shown here that it controls many aspects of *T. trilobus* calcification and positioning. Salinity may influence the vertical distribution of planktonic foraminifera indirectly by changing the density structure of the water column and thereby restricting vertical movement and the accumulation of nutrients at certain depths [78]. It has been observed that in the Atlantic, increases in nutrient concentrations are related to higher salinities [80]. In an effort to attain these high salinity/high nutrient density horizons, *T. trilobus* adjusts its shell weight accordingly.

Through the complex and highly dynamic current system (figure 1), nutrients are transported through intermediate waters from the extratropical regions to the thermocline of the equatorial Atlantic and upwell along the Equator in the Atlantic equatorial divergence. In the vicinity of the nutricline, indicating the deep chlorophyll maximum (DCM) [81], chlorophyll-*a* concentrations reach a maximum between 40 and 90 m water depths [82]. In the Atlantic, in most cases, the DCM layer is found at 100 m depth and generally follows the pycnocline [82]. Even though the depth of the DCM does not change significantly meridionally [71], it changes from east to west. In the equatorial band, the DCM below the mixed layer progressively shoals from approximately 100 m in the west to approximately 70 m in the east [71,81]. Upwelling decreases away from the Equator, and as a consequence, the DCM deepens. Planktonic foraminifera respond to the distribution of chlorophyll, and high abundances are often associated with the DCM [83–85]. Apparently, in the upwelling areas, such as sites 8 (RC24-10) and 9 (RC24-11), *T. trilobus* becomes lighter potentially to decrease its ACD, as its ACD can shoal from the thermocline to distinctly shallower waters within strong upwelling areas [86].

## 6. Conclusions

Based on surface sediment fossil shells, the present study suggests that salinity and physical oceanographic conditions play a significant role in the distribution and calcification of *Trilobatus trilobus* in the central Atlantic Ocean. The analysis of reflected light microscope images and high-resolution images from μCT acquisition provided valuable information about foraminifera shell biometry and integrity. The carbonate content of *T. trilobus* is primarily influenced by seawater salinity, and *in situ* salinity is correlated with various shell properties, including shell weight, area density, test thickness and test percentage within foraminifera individuals. Furthermore, shell weights also depend on *in situ* water density and, to a lesser extent, carbonate ion concentration.

The density horizon within which *T. trilobus* appears to calcify was found to correlate with the density horizons where salinity maximum waters are found, indicating a close relationship between salinity and the species’ habitat. Moreover, the μCT approach revealed novel information about fossil foraminifera biometry and preservation. The results suggest that in equatorial regions, the species adapts by becoming thinner/lighter and shifting its habitat within the thermocline to shallower waters, aligning with the patterns of subtropical cell circulation. These findings support the notion that since foraminifera have active mechanisms to control the calcite saturation state internally, changes in inorganic carbon precipitation for shell formation may be driven by physical oceanographic conditions for buoyancy control and depth habitat adjustments, leading to strong correlations with salinity and water density.

## Data Availability

The Argo Data on which the temperature to calcification depth conversion was based are given in electronic supplementary material, table 1 [87]. The data necessary to reproduce each figure are given in electronic supplementary material, table 3. The high-resolution microcomputed tomographic dataset generated and analysed during this study for Trilobatus trilobus can be found in the Dryad data repository [88].

## References

[1] Kucera M. 2007 Planktonic foraminifera as tracers of past oceanic environments. In Developments in marine geology (ed. C Hillaire–Marcel), pp. 213–262, vol. 1. Amsterdam, The Netherlands: Elsevier. (10.1016/S1572-5480(07)01011-1)

[2] Schiebel R, Hemleben C. 2017 Planktic foraminifers in the modern ocean. Berlin, Germany: Springer. (10.1007/978-3-662-50297-6)

[3] Schiebel R. 2002 Planktic foraminiferal sedimentation and the marine calcite budget. Global Biogeochem. Cycles **16**, 1065. (10.1029/2001GB001459)

[4] Jonkers L, Kučera M. 2017 Quantifying the effect of seasonal and vertical habitat tracking on planktonic foraminifera proxies. Clim. Past **13**, 573–586. (10.5194/cp-13-573-2017)

[5] Zarkogiannis SD, Iwasaki S, Rae JWB, Schmidt MW, Mortyn PG, Kontakiotis G, Hertzberg JE, Rickaby REM. 2022 Calcification, dissolution and test properties of modern planktonic foraminifera from the central Atlantic Ocean. Front. Mar. Sci. **9**. (10.3389/fmars.2022.864801)

[6] Tolderlund DS, Bé AWH, Be AWH. 1971 Seasonal distribution of planktonic foraminifera in the western North Atlantic. Micropaleontol. **17**, 297. (10.2307/1485143)

[7] Lessa D, Morard R, Jonkers L, Venancio IM, Reuter R, Baumeister A, Albuquerque AL, Kucera M. 2020 Distribution of planktonic foraminifera in the subtropical South Atlantic: depth hierarchy of controlling factors. Biogeosciences **17**, 4313–4342. (10.5194/bg-17-4313-2020)

[8] Spezzaferri S, Kucera M, Pearson PN, Wade BS, Rappo S, Poole CR, Morard R, Stalder C. 2015 Fossil and genetic evidence for the polyphyletic nature of the planktonic foraminifera 'Globigerinoides', and description of the new genus Trilobatus. PLoS One **10**, e0128108. (10.1371/journal.pone.0128108) 26020968 PMC4447400

[9] André A *et al*. 2013 The cryptic and the apparent reversed: lack of genetic differentiation within the morphologically diverse plexus of the planktonic foraminifer Globigerinoides sacculifer. Palaeobiology **39**, 21–39. (10.1666/0094-8373-39.1.21)

[10] Wood M, Hayes CT, Paytan A. 2023 Global quaternary carbonate burial: proxy- and model-based reconstructions and persisting uncertainties. Ann. Rev. Mar. Sci. **15**, 277–302. (10.1146/annurev-marine-031122-031137) 35773213

[11] Prell WL, Martin A, Cullen JL, Trend M. 1999 The Brown University foraminiferal data base. NOAA National Centers for Environmental Information. (10.25921/WMEJ-6P14)

[12] Hemleben C, Anderson OR, Spindler M. 1989 Modern planktonic foraminifera. New York, NY: Springer-Verlag. (10.1007/978-1-4612-3544-6) 17813291

[13] Schiebel R, Hemleben C. 2005 Modern planktic foraminifera. Paläontol. Z. **79**, 135–148. (10.1007/BF03021758)

[14] Cléroux C, deMenocal P, Arbuszewski J, Linsley B. 2013 Reconstructing the upper water column thermal structure in the Atlantic Ocean. Palaeoceanography **28**, 503–516. (10.1002/palo.20050)

[15] Dai Y, Yu J, deMenocal P, Hyams‐Kaphzan O. 2019 Influences of temperature and secondary environmental parameters on planktonic foraminiferal Mg/Ca: a new core‐top calibration. Geochem. Geophys. Geosyst. **20**, 4370–4381. (10.1029/2019GC008526)

[16] Broecker W, Barker S, Clark E, Hajdas I, Bonani G. 2006 Anomalous radiocarbon ages for foraminifera shells. Palaeoceanography **21**. (10.1029/2005PA001212)

[17] Zhang D, McPhaden MJ, Johns WE. 2003 Observational evidence for flow between the subtropical and tropical Atlantic: the Atlantic subtropical cells. J. Phys. Oceanogr. **33**, 1783–1797. (10.1175/2408.1)

[18] Schott FA, Fischer J, Stramma L. 1998 Transports and pathways of the upper-layer circulation in the Western Tropical Atlantic. J. Phys. Oceanogr. **28**, 1904–1928. (10.1175/1520-0485(1998)028<1904:TAPOTU>2.0.CO;2)

[19] Ravelo AC, Fairbanks RG. 1992 Oxygen isotopic composition of multiple species of planktonic foraminifera: recorders of the modern photic zone temperature gradient. Paleoceanography **7**, 815–831. (10.1029/92PA02092)

[20] Stramma L, Schott F. 1999 The mean flow field of the tropical Atlantic Ocean. Deep Sea Res. Top. Stud. Oceanogr. **46**, 279–303. (10.1016/S0967-0645(98)00109-X)

[21] Bauer E, Siedler G. 1988 The relative contributions of advection and isopycnal and diapycnal mixing below the subtropical salinity maximum. Deep Sea Res. A. Oceanogr. Res. Pap. **35**, 811–837. (10.1016/0198-0149(88)90032-5)

[22] Tsuchiya M, Talley LD, McCartney MS. 1992 An eastern Atlantic section from Iceland southwards across the equator. Deep Sea Res. A. Oceanogr. Res. Pap. **39**, 1885–1917. (10.1016/0198-0149(92)90004-D)

[23] Tomczak M, Godfrey JS. 1994 Regional oceanography: introduction. New York, NY: Elsevier.

[24] Carlson AE, Oppo DW, Came RE, LeGrande AN, Keigwin LD, Curry WB. 2008 Subtropical Atlantic salinity variability and Atlantic meridional circulation during the last deglaciation. Geology **36**, 991. (10.1130/G25080A.1)

[25] Shcherbina AY, D’Asaro EA, Riser SC, Kessler WS. 2015 Variability and interleaving of upper-ocean water masses surrounding the north Atlantic salinity maximum. Oceanography **28**, 106–113. (10.5670/oceanog.2015.12)

[26] Mollenhauer G, Grotheer H, Gentz T, Bonk E, Hefter J. 2021 Standard operation procedures and performance of the MICADAS radiocarbon laboratory at Alfred Wegener Institute (AWI), Germany. Nucl. Instrum. Methods Phys. Res. B **496**, 45–51. (10.1016/j.nimb.2021.03.016)

[27] Berger WH, Bonneau MC, Parker FL. 1982 Foraminifera on the deep-sea floor: lysocline and dissolution rate. Oceanol. Acta **5**, 249–258. https://archimer.ifremer.fr/doc/00120/23161/21006.pdf

[28] Poole CR, Wade BS. 2019 Systematic taxonomy of the Trilobatus sacculifer plexus and descendant Globigerinoidesella fistulosa (planktonic foraminifera). J. Syst. Palaeontol. **17**, 1989–2030. (10.1080/14772019.2019.1578831)

[29] de Villiers S, Greaves M, Elderfield H. 2002 An intensity ratio calibration method for the accurate determination of Mg/Ca and Sr/Ca of marine carbonates by ICP‐AES. Geochem. Geophys. Geosyst. **3**. (10.1029/2001GC000169)

[30] Beer CJ, Schiebel R, Wilson PA. 2010 Technical note: on methodologies for determining the size-normalized weight of planktic foraminifera. Biogeosciences **7**, 2193–2198. (10.5194/bg-7-2193-2010)

[31] Mulqueeney JM, Searle-Barnes A, Brombacher A, Sweeney M, Goswami A, Ezard THG. 2024 How many specimens make a sufficient training set for automated three-dimensional feature extraction? R. Soc. Open Sci. **11**, rsos.240113. (10.1098/rsos.240113) 39100182 PMC11296157

[32] Marshall BJ, Thunell RC, Henehan MJ, Astor Y, Wejnert KE. 2013 Planktonic foraminiferal area density as a proxy for carbonate ion concentration: a calibration study using the Cariaco Basin ocean time series. Palaeoceanography **28**, 363–376. (10.1002/palo.20034)

[33] Zarkogiannis SD, Fernandez V, Greaves M, Mortyn PG, Kontakiotis G, Antonarakou A. 2020 X-ray tomographic data of planktonic foraminifera species Globigerina bulloides from the Eastern Tropical Atlantic across termination II. GigaByte **2020**, gigabyte5. (10.46471/gigabyte.5) 36824589 PMC9632000

[34] Iwasaki S, Kimoto K, Sasaki O, Kano H, Honda MC, Okazaki Y. 2015 Observation of the dissolution process of Globigerina bulloides tests (planktic foraminifera) by X‐ray microcomputed tomography. Palaeoceanography **30**, 317–331. (10.1002/2014PA002639)

[35] Choquel C, Müter D, Ni S, Pirzamanbein B, Charrieau LM, Hirose K, Seto Y, Schmiedl G, Filipsson HL. 2023 3D morphological variability in foraminifera unravel environmental changes in the Baltic Sea entrance over the last 200 years. Front. Earth Sci. **11**. (10.3389/feart.2023.1120170)

[36] Argo. 2000 Argo float data and metadata from Global Data Assembly Centre (Argo GDAC). SEANOE (10.17882/42182)

[37] Be AWH. 1960 Ecology of recent planktonic foraminifera: part 2: bathymetric and seasonal distributions in the Sargasso Sea off Bermuda. Micropaleontol. **6**, 373. (10.2307/1484218)

[38] Key RM *et al*. 2004 A global ocean carbon climatology: results from global data analysis project (GLODAP). Global Biogeochem. Cycles **18**. (10.1029/2004GB002247)

[39] Heuven S, Pierrot D, Rae JWB, Lewis E, Wallace DWR. 2011 CO2SYS v782 1.1. Oak Ridge, TN: Oak Ridge National Laboratory.

[40] Schlitzer R. 2002 Interactive analysis and visualization of geoscience data with ocean data view. Comput. Geosci. **28**, 1211–1218. (10.1016/S0098-3004(02)00040-7)

[41] Barker S, Greaves M, Elderfield H. 2003 A study of cleaning procedures used for foraminiferal Mg/Ca palaeothermometry. Geochem. Geophys. Geosyst. **4**. (10.1029/2003GC000559)

[42] Greaves M *et al*. 2008 Interlaboratory comparison study of calibration standards for foraminiferal Mg/Ca thermometry. Geochem. Geophys. Geosyst. **9**. (10.1029/2008GC001974)

[43] Regenberg M, Steph S, Nürnberg D, Tiedemann R, Garbe-Schönberg D. 2009 Calibrating Mg/Ca ratios of multiple planktonic foraminiferal species with δ^18^O-calcification temperatures: palaeothermometry for the upper water column. Earth Planet. Sci. Lett. **278**, 324–336. (10.1016/j.epsl.2008.12.019)

[44] Hertzberg JE, Schmidt MW. 2013 Refining Globigerinoides ruber Mg/Ca palaeothermometry in the Atlantic Ocean. Earth Planet. Sci. Lett. **383**, 123–133. (10.1016/j.epsl.2013.09.044)

[45] Kimoto K, Horiuchi R, Sasaki O, Iwashita T. 2023 Precise bulk density measurement of planktonic foraminiferal test by X-ray microcomputed tomography. Front. Earth Sci. **11**. (10.3389/feart.2023.1184671)

[46] Ujié H. 1968 Distribution of living planktonic foraminifera in the southeast Indian Ocean. Bull. Natl. Sci. Mus. Tokyo **11**, 98–125.

[47] Gómez-Morales J, Torrent-Burgués J, López-Macipe A, Rodríguez-Clemente R. 1996 Precipitation of calcium carbonate from solutions with varying ratios. J. Cryst. Growth **166**, 1020–1026. (10.1016/0022-0248(96)00083-8)

[48] Roger R 1934 Physico-chemical factors affecting the solubility of calcium carbonate in sea water. SEPM J. Sediment. Res. **4**, 103–110. (10.1306/D4268ED2-2B26-11D7-8648000102C1865D)

[49] Lombard F, da Rocha RE, Bijma J, Gattuso JP. 2010 Effect of carbonate ion concentration and irradiance on calcification in planktonic foraminifera. Biogeosciences **7**, 247–255. (10.5194/bg-7-247-2010)

[50] Dong S, Lei Y, Bi H, Xu K, Li T, Jian Z. 2022 Biological response of planktic foraminifera to decline in seawater pH. Biology **11**, 98. (10.3390/biology11010098) 35053097 PMC8773009

[51] Bakker DCE, de Baar HJW, de Jong E. 1999 The dependence on temperature and salinity of dissolved inorganic carbon in East Atlantic surface waters. Mar. Chem. **65**, 263–280. (10.1016/S0304-4203(99)00017-1)

[52] Toyofuku T *et al*. 2017 Proton pumping accompanies calcification in foraminifera. Nat. Commun. **8**, 14145. (10.1038/ncomms14145) 28128216 PMC5290161

[53] de Nooijer LJ, Toyofuku T, Kitazato H. 2009 Foraminifera promote calcification by elevating their intracellular pH. Proc. Natl Acad. Sci. USA **106**, 15 374–15 378. (10.1073/pnas.0904306106) PMC2741258 19706891

[54] Hönisch B *et al*. 2013 The influence of salinity on Mg/Ca in planktic foraminifers – evidence from cultures, core-top sediments and complementary δ^18^O. Geochim. Cosmochim. Acta **121**, 196–213. (10.1016/j.gca.2013.07.028)

[55] Kısakürek B, Eisenhauer A, Böhm F, Garbe-Schönberg D, Erez J. 2008 Controls on shell Mg/Ca and Sr/Ca in cultured planktonic foraminiferan, Globigerinoides ruber (white). Earth Planet. Sci. Lett. **273**, 260–269. (10.1016/j.epsl.2008.06.026)

[56] Lea DW, Mashiotta TA, Spero HJ. 1999 Controls on magnesium and strontium uptake in planktonic foraminifera determined by live culturing. Geochim. Cosmochim. Acta **63**, 2369–2379. (10.1016/S0016-7037(99)00197-0)

[57] Hauzer H, Evans D, Müller W, Rosenthal Y, Erez J. 2021 Salinity effect on trace element incorporation in cultured shells of the large benthic foraminifer Operculina ammonoides. Palaeoceanogr. Palaeoclimatol. **36**, e2021PA004218. (10.1029/2021PA004218)

[58] Gray WR, Evans D, Henehan M, Weldeab S, Lea DW, Müller W, Rosenthal Y. 2023 Sodium incorporation in foraminiferal calcite: an evaluation of the Na/Ca salinity proxy and evidence for multiple Na-bearing phases. Geochim. Cosmochim. Acta **348**, 152–164. (10.1016/j.gca.2023.03.011)

[59] Dueñas-Bohórquez A, da Rocha RE, Kuroyanagi A, Bijma J, Reichart GJ. 2009 Effect of salinity and seawater calcite saturation state on Mg and Sr incorporation in cultured planktonic foraminifera. Mar. Micropaleontol. **73**, 178–189. (10.1016/j.marmicro.2009.09.002)

[60] Branson O, Redfern SAT, Tyliszczak T, Sadekov A, Langer G, Kimoto K, Elderfield H. 2013 The coordination of Mg in foraminiferal calcite. Earth Planet. Sci. Lett. **383**, 134–141. (10.1016/j.epsl.2013.09.037)

[61] Marriott CS, Henderson GM, Crompton R, Staubwasser M, Shaw S. 2004 Effect of mineralogy, salinity, and temperature on Li/Ca and Li isotopic composition of calcium carbonate. Chem. Geol. **212**, 5–15. (10.1016/j.chemgeo.2004.08.002)

[62] Okumura M, Kitano Y. 1986 Coprecipitation of alkali metal ions with calcium carbonate. Geochim. Cosmochim. Acta **50**, 49–58. (10.1016/0016-7037(86)90047-5)

[63] Zarkogiannis SD, Antonarakou A, Tripati A, Kontakiotis G, Mortyn PG, Drinia H, Greaves M. 2019 Influence of surface ocean density on planktonic foraminifera calcification. Sci. Rep. **9**, 533. (10.1038/s41598-018-36935-7) 30679608 PMC6346091

[64] Marszalek DS. 1982 The role of heavy skeletons in vertical movements of nonmotile zooplankton. Mar. Behav. Physiol. **8**, 295–303. (10.1080/10236248209387026)

[65] Johnstone HJH, Schulz M, Barker S, Elderfield H. 2010 Inside story: an X-ray computed tomography method for assessing dissolution in the tests of planktonic foraminifera. Mar. Micropaleontol. **77**, 58–70. (10.1016/j.marmicro.2010.07.004)

[66] Sadekov AY, Eggins SM, De Deckker P. 2005 Characterization of Mg/Ca distributions in planktonic foraminifera species by electron microprobe mapping. Geochem. Geophys. Geosyst. **6**. (10.1029/2005GC000973)

[67] Arbuszewski J, deMenocal P, Kaplan A, Farmer EC. 2010 On the fidelity of shell-derived δ^18^O_seawater_ estimates. Earth Planet. Sci. Lett. **300**, 185–196. (10.1016/j.epsl.2010.10.035)

[68] Ofstad S, Zamelczyk K, Kimoto K, Chierici M, Fransson A, Rasmussen TL. 2021 Shell density of planktonic foraminifera and pteropod species Limacina helicina in the Barents Sea: relation to ontogeny and water chemistry. PLoS One **16**, e0249178. (10.1371/journal.pone.0249178) 33909623 PMC8081242

[69] Farmer EC, Kaplan A, de Menocal PB, Lynch-Stieglitz J. 2007 Corroborating ecological depth preferences of planktonic foraminifera in the tropical Atlantic with the stable oxygen isotope ratios of core top specimens. Paleoceanography **22**, PA3205. (10.1029/2006PA001361)

[70] Rebotim A, Voelker AHL, Jonkers L, Waniek JJ, Meggers H, Schiebel R, Fraile I, Schulz M, Kucera M. 2016 Factors controlling the depth habitat of planktonic foraminifera in the subtropical eastern North Atlantic. Paleobiogeo. 1–48. (10.5194/bg-2016-348)

[71] Wang X, Murtugudde R, Hackert E, Marañón E. 2013 Phytoplankton carbon and chlorophyll distributions in the equatorial Pacific and Atlantic: a basin-scale comparative study. J. Mar. Syst. **109–110**, 138–148. (10.1016/j.jmarsys.2012.03.004)

[72] Sverdrup HU, Johnson MW, Fleming RH. 1942 The oceans: their physics, chemistry, and general biology. New York, NY: Prentice-Hall, Inc.

[73] Simstich J, Sarnthein M, Erlenkeuser H. 2003 Paired δ^18^O signals of Neogloboquadrina pachyderma (s) and Turborotalita quinqueloba show thermal stratification structure in Nordic Seas. Mar. Micropaleontol. **48**, 107–125. (10.1016/S0377-8398(02)00165-2)

[74] Stainbank S, Kroon D, Rüggeberg A, Raddatz J, de Leau ES, Zhang M, Spezzaferri S. 2019 Controls on planktonic foraminifera apparent calcification depths for the northern equatorial Indian Ocean. PLoS One **14**, e0222299. (10.1371/journal.pone.0222299) 31513624 PMC6767952

[75] Rickaby REM, Halloran P. 2005 Cool La Niña during the warmth of the Pliocene? Science **307**, 1948–1952. (10.1126/science.1104666) 15790852

[76] Schott FA, Mccrear JP, Johnson GC. 2004 Shallow overturning circulations of the tropical-subtropical oceans. In Earth’s climate (eds C Wang, S Xie, J Carton), pp. 261–304. (10.1029/147GM15)

[77] Malanotte-Rizzoli P, Hedstrom K, Arango H, Haidvogel DB. 2000 Water mass pathways between the subtropical and tropical ocean in a climatological simulation of the North Atlantic ocean circulation. Dyn. Atmos. Ocean. **32**, 331–371. (10.1016/S0377-0265(00)00051-8)

[78] Bijma J, Faber WW, Hemleben C. 1990 Temperature and salinity limits for growth and survival of some planktonic foraminifers in laboratory cultures. J. Foram. Res. **20**, 95–116. (10.2113/gsjfr.20.2.95)

[79] Mulitza S, Wolff T, Pätzold J, Hale W, Wefer G. 1998 Temperature sensitivity of planktic foraminifera and its influence on the oxygen isotope record. Mar. Micropaleontol. **33**, 223–240. (10.1016/S0377-8398(97)00040-6)

[80] van Aken HM. 2001 The hydrography of the mid-latitude Northeast Atlantic Ocean — Part III: the subducted thermocline water mass. Deep Sea Res. I: Oceanogr. Res. Papers **48**, 237–267. (10.1016/S0967-0637(00)00059-5)

[81] Herbland A, Le Bouteiller A, Raimbault P. 1985 Size structure of phytoplankton biomass in the equatorial Atlantic Ocean. Deep Sea Res. A **32**, 819–836. (10.1016/0198-0149(85)90118-9)

[82] Vedernikov VI, Gagarin VI, Demidov AB, Burenkov VI, Stunzhas PA. 2007 Primary production and chlorophyll distributions in the subtropical and tropical waters of the Atlantic Ocean in the autumn of 2002. Oceanol. **47**, 386–399. (10.1134/S0001437007030113)

[83] Fairbanks RG, Sverdlove M, Free R, Wiebe PH, Bé AWH. 1982 Vertical distribution and isotopic fractionation of living planktonic foraminifera from the Panama Basin. Nature **298**, 841–844. (10.1038/298841a0)

[84] Mortyn PG, Charles CD. 2003 Planktonic foraminiferal depth habitat and δ^18^o calibrations: plankton tow results from the atlantic sector of the southern ocean. Paleoceanography **18**. (10.1029/2001PA000637)

[85] Schiebel R, Waniek J, Bork M, Hemleben C. 2001 Planktic foraminiferal production stimulated by chlorophyll redistribution and entrainment of nutrients. Deep-Sea Res. I **48**, 721–740. (10.1016/S0967-0637(00)00065-0)

[86] Loubere P. 2001 Nutrient and oceanographic changes in the Eastern Equatorial Pacific from the last full glacial to the present. Glob. Planet. Change **29**, 77–98. (10.1016/S0921-8181(00)00085-0)

[87] Zarkogiannis SD. 2024 Supplementary material from: Calcification and ecological depth preferences of the planktonic foraminifer trilobatus trilobus in the central atlantic. Figshare. (10.6084/m9.figshare.c.7501103) PMC11615192 39635149

[88] Zarkogiannis SD. 2024 Tomographic data of Trilobatus trilobus shells from central Atlantic core-top sediment samples [dataset]. Dryad. (10.5061/dryad.6t1g1jx6q)

